# Supporting data for comparative proteomic analysis of *Listeria monocytogenes* ATCC 7644 exposed to a sublethal concentration of nisin

**DOI:** 10.1016/j.dib.2015.02.013

**Published:** 2015-02-26

**Authors:** Kendi Nishino Miyamoto, Karina Mariante Monteiro, Karin da Silva Caumo, Karina Rodrigues Lorenzatto, Henrique Bunselmeyer Ferreira, Adriano Brandelli

**Affiliations:** a Programa de Pós-Graduação em Biologia Celular e Molecular, Centro de Biotecnologia, Universidade Federal do Rio Grande do Sul (UFRGS), Porto Alegre, Brazil; b Laboratório de Genômica Estrutura e Funcional, Centro de Biotecnologia, UFRGS, Porto Alegre, Brazil; c Departamento de Biologia Molecular e Biotecnologia, Instituto de Biociências, UFRGS, Porto Alegre, Brazil; d Laboratório de Bioquímica e Microbiologia Aplicada, Instituto de Ciência e Tecnologia de Alimentos, UFRGS, Porto Alegre, Brazil

## Abstract

Here we provide the LC–MS/MS data from a comparative analysis of *Listeria monocytogenes* ATCC 7644 treated and non-treated with a sublethal concentration of nisin (10^−3^ mg/mL). Protein samples were analyzed by multidimensional protein identification technology (MudPIT) approach, in an off-line configuration. The raw MS/MS data allowed the detection of 49,591 spectra which resulted in 576 protein identifications. After Scaffold validation, 179 proteins were identified with high confidence. A label-free quantitative analysis based of normalized spectral abundance factor (NSAF) was used and 13 proteins were found differentially expressed between nisin-treated and non-treated cells. Gene ontology analysis of differentially expressed proteins revealed that most of them are correlated to metabolic process, oxidative stress response mechanisms and molecular binding. A detailed analysis and discussion of these data may be found in Miyamoto et al. [1].

## Specifications table

**Table:** 

Subject area	Biology

More specific subject area	Proteomics and microbiology
Type of data	Tables
How data was acquired	MudPIT approach consisting in an offline strong cation exchange (SCX) fractionation step associated with LC–MS/MS analysis using a Q-Tof Premier API mass spectrometer (MicroMass/Waters) coupled to a nano-UPLC (Reverse-phase-UPLC, nanoAcquity/Waters).
Data format	Analyzed (Table D1) and Validated (Table D2)
	MS/MS raw data were processed using Mascot Distiller 2.2.1 and database search was performed by Mascot Search engine 2.3.0. Mascot results were analyzed, validated, quantitatively assessed and statistically evaluated by Scaffold Q+ 4.4.1.1. Gene ontology terms were assigned by Blast2GO 3.0.7.
Experimental factors	Bacterial cells were lysed by sonication and proteins were solubilized with Rapigest surfactant, reduced with DTT and alkylated with iodoacetamide, followed by trypsin digestion.
Experimental features	Digested proteins from L. monocytogenes ATCC 7644 nisin-treated and non-treated cells were fractioned by off-line SCX spin columns, desalted and analyzed by LC–MS–MS in an RP-UPLC coupled to ESI-Q-ToF mass spectrometer.
Data source location	Porto Alegre, Brazil and Campinas, Brazil
Data accessibility	Data are present with this article

## Value of the data

• First proteomic profiles of the *L. monocytogenes* pathogenic strain ATCC 7644, with the high confidence identification of proteins related with several biological processes.
• Proteins identified in experimental conditions with or without nisin provide more insights of *L. monocytogenes* ATCC 7644 molecular responses triggered by this bacteriocin.
• Functional annotation of the differentially expressed proteins in response to nisin indicates their involvement with metabolic processes, oxidative stress response mechanisms and molecular binding.

## 1 Data, experimental design, materials and methods

### 1.1 Data

Supplementary Table 1. Overview of MS data and protein/peptide identification reports of nisin-treated and non-treated *L. monocytogenes* ATCC 7644 samples.

Supplementary Table 2. Scaffold validated protein identifications, quantitative analysis and GO functional annotation of nisin-treated and non-treated control *L. monocytogenes* ATCC 7644 samples.

### 1.2 Experimental design

*L. monocytogenes* ATCC 7644 cultures were treated with 10^−3^ mg/mL of nisin during mid-log growth phase and cells were collected after 1 h post bacteriocin inoculation. Non-treated cells were used as control samples. Bacterial protein extracted were trypsin digested and submitted to MudPIT analysis using an off-line SCX fractionation step. SCX fractions were desalted and analyzed by LC–MS/MS. MS/MS data were processed and analyzed by MASCOT platform. Proteomic data were validated and comparatively analyzed by Scaffold Q+ [1].

## 2 Material and methods

### 2.1 Protein extraction

Bacterial cells from of 10^−3^ mg/mL nisin-treated and untreated control cultures were recovered during the mid-log growth phase, 60 min after treatment (10 mM HCl solution was applied to non-treated control cells). The samples were centrifuged (4500*g*) and the cell pellets were washed five times with 20 mM Tris–HCl pH 8.0. Cell suspensions were sonicated in an ice bath for 6 cycles (Vibra Cell™ VC601, Sonics and Materials Inc., Newtown, CT, USA) of 30 s with a 60 s interval between each cycle. The resultant protein extracts were quantified using the fluorometric *Qubit*^*®*^ *Protein Assay* kit (Invitrogen/Life Technologies, Grand Island, NY, USA), read in a *Qubit*^*®*^2.0 *fluorometer* (Invitrogen/Life Technologies) and stored at −20 ^o^C. Two replicates for each condition, named CA and CB (for non-treated controls) and TA and TB (for nisin-treated samples), were used for proteomic experiments.

### 2.2 Sample preparation for mass spectrometry

The protein extracts were solubilized with 0.1% (w/v) RapiGest^®^ SF surfactant (Waters, Milford, MA, USA) in 20 mM Tris–HCl, pH 8.0. Proteins were reduced and alkylated with 5 mM dithiothreitol (DTT) (Bio-Rad, Hercules, CA, USA) and 15 mM iodoacetamide (Bio-Rad), respectively. Proteins were digested with Trypsin Gold Mass Spectrometry Grade (Promega, Madison, WI, USA) at a ratio of 1 µg of enzyme per 50 µg of the protein and incubated overnight at 37 °C.

RapiGest was removed from samples with 0.5% trifluoroacetic acid (TFA) followed by incubation at 37 °C for 45 min and centrifugation at 13,000*g* for 10 min. Digested samples were dried in a vacuum concentrator (miVac DNA concentrator, GenVac, Ipswich, UK) and stored at −20 °C. Peptides were suspended with 0.1% TFA and desalted using a reversed-phase column (Oasis HLB Cartridge, Waters). The eluted peptides were dried in a vacuum concentrator suspended in 5 mM KH_2_PO_4_/25% acetonitrile, pH 3.0.

Peptides were submitted to a multidimensional protein identification technology (MudPIT) approach [2] with an offline strong cationic exchange (SCX) step using a PolySULFOETHYL Aspartamide^TM^ SCX Minispin Column (Harvard Apparatus, Holliston, MA, USA), and eluted in 5 fractions with 20, 40, 60, 80 and 500 mM KCl. Desalting of each SCX fraction was performed in a C18 reversed-phase Minispin Column (Harvard Apparatus) and samples were dried in a vacuum concentrator and stored at −20 °C until LC–MS/MS analysis.

### 2.3 LC–MS/MS analysis

Peptides of each SCX fraction from test and control samples were separately analyzed using a Q-Tof Premier API mass spectrometer (MicroMass/Waters), attached to a nanoACQUITY^TM^ ultra performance liquid chromatography (UPLC) system (Waters). Ten micrograms of each peptide sample were injected in an analytic ACQUITY UPLC peptide BEH C18 nanoAcquity column (130 Å, 1.7 µm, 100 µm×100 mm) with a 2–90% acetonitrile gradient in 0.1% formic acid for 60 min, at a 200 nL/min flow rate. An ACQUITY UPLC Symmetry C18 nanoACQUITY trap column (100 Å, 5 μm, 180 μm×20 mm) was used for sample desalting at a flow rate of 5 µl/min over 2 min. The MS spectra between *m/z* 100 and 2000 Da were recorded, with 1-second search time spaced by 0.1 s interval. The MS/MS spectra was acquired on *m/z* 50–2000 Da mass range with the same search time and interval mentioned for the MS mode, using the MassLynx software system (Waters). The samples were analyzed at data dependent acquisition mode, in which every full MS mode run was followed by three consecutive MS/MS runs of the three most intense multiple charged ions with a count higher than the threshold (30 counts/s). The collision energy values necessary for the peptide fragmentation were adjusted according to the +2, +3 and +4 ion charges recognition files available by the MassLynx system. The raw MS/MS data were processed using the Mascot Distiller v. 2.2.1 (Matrix Science, Boston, MA, USA) to generate the ⁎.mgf peak list files. Each SCX fraction from test and control samples was run twice (LC–MS/MS technical replicates).

### 2.4 MS/MS data analysis

The database for protein search comprises deduced amino acid sequences (2909 entries) from *L. monocytogenes* FSL R2-561 strain (which belongs to the same 1/2c serotype as the ATCC 7644 strain used in the experiments), available at UniProtKB website (http://www.uniprot.org/taxonomy/393126, proteome ID UP000001287, last update: 28/10/2014). Common contaminants (such as human keratin and porcine trypsin) were also included in the database ⁎.fasta file, in order to avoid misidentifications. The MS/MS peak list data files were analyzed by Mascot ion search engine version 2.3.0, using carbamidomethylation of cysteine as a fixed modification (monoisotopic mass 57.0215 Da), methionine oxidation as a variable modification (monoisotopic mass 15.9949 Da), and a peptide and MS/MS fragment ion mass tolerance of 0.1 Da. Other parameters were set to include up to one missed cleavage, and the Mascot automatic decoy database search was selected. The MASCOT DAT files from all SCX fractions of each biological replica (CA, CB, TA and TB) were merged (including the LC–MS/MS technical replicates) and assembled by Scaffold Q+ version 4.4.1.1 (Proteome Software, Portland, OR, USA) to generate a full report of proteomic data, which is provided in Supplementary Table 1. Protein identification validation was performed by Scaffold parameters including Mascot ion scores of 30 or higher (for +2, +3 and +4 charges), a minimum of two identified peptides, parent mass tolerance of 100 ppm, 90% peptide identification probability (using the Scaffold Local FDR algorithm), and 99% protein identification probability (using the Protein Prophet algorithm [3]), resulting in a 0.0% decoy FDR. The normalized spectral abundance factor (NSAF) [4] was calculated for each protein, and quantitative differences were statistically analyzed by a *t*-test using Scaffold Q+ version 4.4.1.1. Differences with *p* values lower than 0.05 were considered statistically significant. Identified proteins were categorized according to gene ontology terms using the software Blast2GO version 3.0.7 (BioBam, Valencia, Spain) [5]. Scaffold validated identified proteins, abundance values and comparative analysis between control and nisin-treated samples are shown in Supplementary Table 2. This table also contains the GO functional categories assigned to the identified proteins.
